# Predicting diabetes second-line therapy initiation in the Australian population via time span-guided neural attention network

**DOI:** 10.1371/journal.pone.0211844

**Published:** 2019-10-18

**Authors:** Samuele Fiorini, Farshid Hajati, Annalisa Barla, Federico Girosi

**Affiliations:** 1 Iren S.p.A, Genoa, Italy; 2 School of Information Technology and Engineering, MIT Sydney, Sydney, New South Wales, Australia; 3 Translational Health Research Institute, Western Sydney University, Penrith, New South Wales, Australia; 4 Capital Markets CRC, Sydney, New South Wales, Australia; 5 Department of Informatics, Bioengineering, Robotics and System Engineering (DIBRIS), University of Genoa, Genoa, Italy; 6 Digital Health CRC, Sydney, New South Wales, Australia; Northwestern Polytechnical University, CHINA

## Abstract

**Introduction:**

The first line of treatment for people with Diabetes mellitus is metformin. However, over the course of the disease metformin may fail to achieve appropriate glycemic control, and a second-line therapy may become necessary. In this paper we introduce Tangle, a time span-guided neural attention model that can accurately and timely predict the upcoming need for a second-line diabetes therapy from administrative data in the Australian adult population. The method is suitable for designing automatic therapy review recommendations for patients and their providers without the need to collect clinical measures.

**Data:**

We analyzed seven years of de-identified records (2008-2014) of the 10% publicly available linked sample of Medicare Benefits Schedule (MBS) and Pharmaceutical Benefits Scheme (PBS) electronic databases of Australia.

**Methods:**

By design, Tangle inherits the representational power of pre-trained word embedding, such as GloVe, to encode sequences of claims with the related MBS codes. Moreover, the proposed attention mechanism natively exploits the information hidden in the time span between two successive claims (measured in number of days). We compared the proposed method against state-of-the-art sequence classification methods.

**Results:**

Tangle outperforms state-of-the-art recurrent neural networks, including attention-based models. In particular, when the proposed time span-guided attention strategy is coupled with pre-trained embedding methods, the model performance reaches an Area Under the ROC Curve of 90%, an improvement of almost 10 percentage points over an attentionless recurrent architecture.

**Implementation:**

Tangle is implemented in Python using Keras and it is hosted on GitHub at https://github.com/samuelefiorini/tangle.

## Introduction

Diabetes mellitus (DM) affects around 1.2 million of Australians aged 2 years and over. In the last two decades, the prevalence of the disease almost doubled, reaching 5.1% of the population in 2015 (Source Australian Government—Department of Health: https://bit.ly/2Njqidp, last visited on January 2019). In the same year, 85% of the Australians with DM reported a Type 2 diagnosis (T2DM). This type of disease is particularly worrisome as it is the leading cause of more than half of the diabetes-related deaths of 2015 [1]. In order to reach glycemic control in T2DM subjects, all the major wordlwide diabetes associations recommend dietary changes and physical exercise along with administration of metformin, if needed [2]. When metformin is not sufficient anymore, second-line medications should be added [3]. Failing to do so will lead to worsening conditions and therefore it is important to identify those patients who should be targeted for therapy change, so they can be monitored closely.

Thanks to recent advances in the field of machine learning it is becoming possible to design algorithms that exploit medical records to predict and identify those patients who may benefit from specific interventions [4].

In this paper we describe a predictive algorithm that, given the administrative medical records history of patients with DM, estimates the likelihood that they will need second-line medication in the next future. This method can be used as a tool to design an automatic system for patients and/or their providers, that notifies them when a change in therapy might be worth considering. From a machine learning point of view this means that the model is a classifier trained on labeled sequences of medical events, where the binary labels identify subjects that added a second-line medication.

The medical events we consider in this paper are any of the events reported for administrative purposes in the Medicare Benefits Schedule (MBS), that records the utilization of primary care services such as visits to GPs and specialists, diagnostic and pathology testing as well as therapeutics procedures. Using actual clinical records seems an appealing, albeit more complex, option and might result in better predictions. However, we have not considered it because an integrated system of health records has not been implemented yet at national level. MBS records, instead, are not only routinely collected at federal level for administrative purposes, but are also, to some extent, available for data analysis.

## Background

In this paper we focus on learning a classification function for sequences, *i.e*. ordered lists of events, that are encoded by symbolic values [5]. A major challenge with this type of data consists in mapping them to a numerical representation suitable to train a classification model. Standard vector representations, adopted for instance in natural language processing, can be either *dense* (*i.e*. most of the elements are different from zero) or *sparse* (*i.e*. with only few nonzero elements). A popular sparse representation method for symbolic elements, or categorical features, is called One-Hot-Encoding (OHE) and it consists in directly mapping each symbolic element to a unique binary vector [6]. Although frequently used, this representation acts at a local level and it is therefore necessary to adopt some feature aggregation policy to achieve a global representation of a given input sequence. Another sparse representation strategy is multidimensional Bag-of-words (BOW), where each dimension represents the number of occurrences of a given *n*-gram in the sequence [7].

Nowadays, *word embeddings* are the most popular dense representation for sequence learning problems. In this approach, to each element **w**_*i*_ of the sequence **s** (*i.e*. word of the document) it is associated a real-valued dense vector $x_{i}\in X$. The semantic vector space X is designed to have “interesting” properties: *e.g*. neighboring vectors may correspond to words having similar meaning or sharing similar contexts. The two most popular word embeddings models proposed in literature are called Word2Vec [8] and Global Vectors for Word Representation (GloVe) [9].

Once a suitable encoding strategy is defined, a machine learning problem can be posed. In this context, standard sequence classification models can be linear, *e.g*. Logistic Regression (LR) and Support Vector Machines [10], or nonlinear, *e.g*. Random Forests [11] and Boosting [12]. These approaches are usually less computationally expensive than deep learning techniques, and they can also be used in combination with feature selection schemes to promote interpretability of the results [13]. However, this class of techniques suffer from a major drawback: *i.e*. their predictive performance is *heavily* influenced by the discriminative power of the adopted sequence representation.

**Fig 1:**
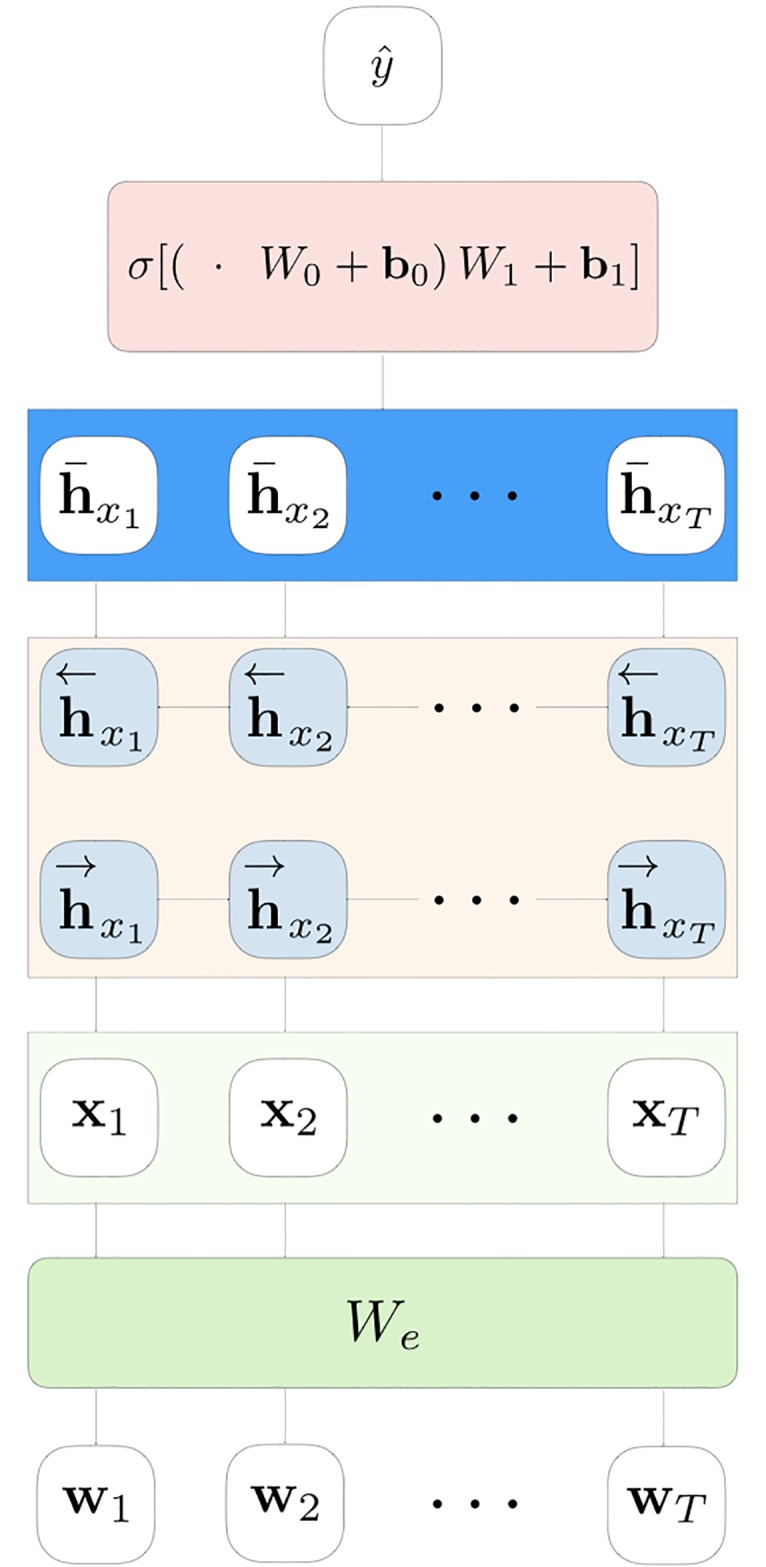
LSTM for sequence classification. A visual representation of a simple bidirectional LSTM for sequences classification. This architecture is used in this work for the sake of comparison, and it is referred to as *baseline*. In this work we adopted LSTM recurrent cells, in order to exploit their ability to learn long-time relationship in the sequences. However, similar architectures can be devised with vanilla RNN, Gated Recurrent Units (GRU) [17] or other types of temporal architectures.

In the recent past, deep learning methods showed remarkable performance in solving complex prediction tasks, such as visual object and speech recognition, image captioning, drug-discovery, *etc* [14]. In the plethora of deep learning models, Recurrent Neural-Networks (RNN) [14] is the class of architectures specifically designed to work with sequential inputs. They consecutively process each element keeping a hidden state vector that can memorize information on the past history. Although designed to learn long-term dependencies, empirical evidence show that vanilla RNN fail in this task. On the other hand, Long Short-Term Memory (LSTM) networks [15], a particular class of RNN, are specifically designed to solve this issue. LSTMs have special memory cells that can work as information accumulator together with a system of input, output and forget gates. These networks empirically showed that they can deal well with both short and long-time relationship among the elements of input sequences. RNN, and deep learning models in general, can also easily inherit the representational power of pre-trained word embeddings, heavily increasing their classification performance [6]. A schematic representation of how RNN-based models can be used to solve a sequence classification task is presented in Fig 1.

Two major shortcomings of these architectures are that: (i) they need to be trained on large data sets, hence requiring high computational time and (ii) when applied in health care-related settings the learned representations hardly align with prior (medical) knowledge [16]. For a comprehensive overview of the most widely adopted deep learning models see [14] and references therein.

Throughout this paper, real-valued variables are indicated with lowercase letters (*e.g*. *a*), one-dimensional vectors with lowercase bold letters (*e.g*. **a**) and matrices, or tensors, with capital letters (*e.g*. *A*). To avoid clutter, sample subscripts are omitted where not strictly needed.

### Neural attention mechanism

Neural attention [18] is a recently proposed strategy to promote interpretability and to improve prediction performance of deep learning methods for document classification [19], machine translation [18] or prediction from sequential Electronic Health Record (EHR) [16, 20, 21]. The intuition behind such attention mechanism is that elements in the sequence have different relevance for the prediction task and that modeling their interactions helps to find the most relevant patterns.

Neural attention mechanism can be seen as a strategy to find *weights* (*α*) that can emphasize events occurring at some point in the sequence, with the final aim to improve the prediction performance. A possible adopted solution to find such weights is via Multi-Layer Perceptron (MLP) [18, 19, 21]. We can summarize the attention mechanism in the next three steps.

$$\begin{array}{c} u_{t}=\text{tanh}(h_{t}W_{t}+b) \end{array}$$ (1)

$$\begin{array}{c} \alpha_{t}=\frac{\text{exp}(u_{t}^{T}w_{\alpha })}{\sum_{t=1}^{T}\text{exp}\left(u_{t}^{T}w_{\alpha }\right)} \end{array}$$ (2)

$$\begin{array}{c} c=\sum_{t=1}^{T}\alpha_{t}h_{t} \end{array}$$ (3)

**Fig 2:**
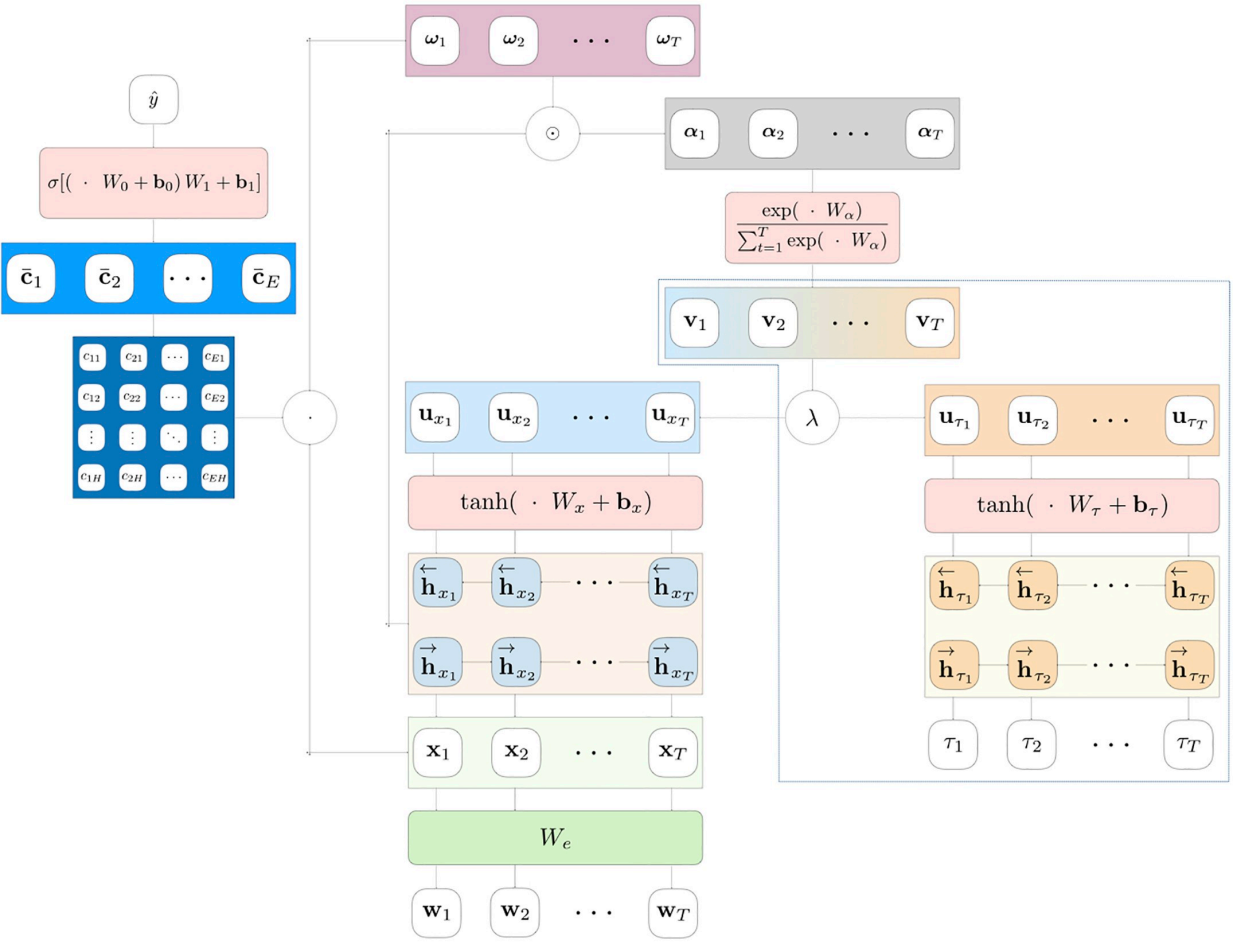
Neural attention model. A visual representation of the attention mechanism for sequences classification. When λ = 1 this corresponds to a standard bidirectional attention model, whereas when λ ≠ 1 the time span sequence *τ*_1_, …, *τ*_*T*_ can guide the model to focus on the most relevant elements of the sequence. We call Tangle the case in which the value of λ is jointly learned during the training process. A blue dashed line highlights the timestamps attention guiding mechanism.

Vectors $h_{t}\in R^{H}$ (for *t* ∈ [1, *T*]) are a sequence of hidden representations obtained by a recurrent architecture from an input sequence of events, such as health service claims or visits. These representations are fed to a one-layer MLP with hyperbolic tangent activation to obtain $u_{t}\in R^{U}$, a hidden representation of **h**_*t*_ (Eq 1). Then, a relevance measure of each element in the sequence (*α*_*t*_) is estimated with a Softmax-activated layer (Eq 2). The weight matrix $W_{t}\in R^{H\times U}$ and the weight vector $w_{\alpha }\in R^{U}$ are jointly learned in the training process. Finally, a context vector **c** can be estimated by computing a weighted sum of the hidden representations **h**_*t*_, with weights *α*_*t*_ (Eq 3). The context vector can then be further transformed by deeper layers, in order to better approximate the target label [19, 20]. A schematic representation of the attention mechanism is summarized in Fig 2.

The use of neural attention models for health-related predictions is extensively explored in literature. In [21] the authors introduce Dipole, a bidirectional recurrent architecture that exploits neural attention to perform sequential EHR forecasting. Differently, in [16] the authors propose GRAM, a graph-based neural attention model that exploits medical ontologies to guide the *α*-estimation step. Whereas, in [20] the authors introduce RETAIN, a neural attention model for prediction from sequential EHR. RETAIN is probably the most relevant work for our purposes. Such model uses two attention levels which separately learn two attention weights vectors that are eventually combined to obtain the context vector. This model achieves good performance when used to predict future diagnosis of heart failure. Although, as the authors claim, it is not capable of exploiting the information hidden in the timestamps of each element of the sequence, which are simply concatenated to each visit embedding (See RETAIN supplemental material [20]).

## Data

We analyzed seven years of de-identified records (2008-2014) of the 10% publicly available linked sample of Medicare Benefits Schedule (MBS) and Pharmaceutical Benefits Scheme (PBS) electronic databases of Australia [22]. MBS-PBS 10% sample data set keeps track of Medicare services subsidized by the Australian government providing information on about 2.1 millions of Australians, who are representative of the full population [23]. The two data sets are linked, meaning that it is possible to track over time the same individual across MBS and PBS claims. MBS-PBS 10% data set also keeps track of other information such as patients’ gender, state of residence and year of birth. PBS data consist of pharmacy transactions for all scripts of drugs of the PBS schedule which are dispensed to individuals holding a Medicare card. In PBS, DM controlling drugs are identified by 90 item codes grouped in two categories: *insulin and analogues* and *blood glucose lowering drugs, excl. insulins*, the latter including metformins. A difficulty that arises when using this data set to extract MBS claims trajectories for a given subject is a rule called *episode coning*. According to it, only the items corresponding to the three most expensive pathologies in an episode of care can be contextually claimed and, therefore, can be extracted from the data set. The rule does not apply to pathology tests requested for hospitalized patients or ordered by specialists.

## Methods

This section provides a detailed definition of the experimental designed followed for the analysis of MBS-PBS 10% data set, as well as an accurate description of model development, validation and comparison.

### Data preprocessing and representation

In this work, we used PBS data to extract the subject IDs corresponding to the population of interest. We first identified all the subjects that make habitual use of DM-controlling pharmaceuticals such as: *Insulins*, *Biguanides* or *Sulfonamides*. From this cohort we identified, and excluded, subjects with gestational diabetes. In order to focus on a stable group of individuals with DM, we included in our analysis only subjects having a concessional card which is used at least for the 75% of the observational years and, in such time interval, for at least 75% of their annual PBS items claims.

Finally, we labeled with *y*_*i*_ = 1 all the subjects that were at first using only Metformin to manage their DM and successively were prescribed to a second-line therapy based on a different drug. This includes both patients that stopped using Metformin at all and patients that associated it with another drug. Conversely, we labeled with *y*_*i*_ = 0 patients that, during the observational time, did not change their Metformin-based DM control therapy. This led us to an imbalanced data set with 26753 subjects which ≈ 22% are positive.

For each subject in our cohort we used the MBS data set to extract the corresponding trajectory of Medicare service claims, which can be represented as the following sequence of tuples

$$\begin{array}{c} \left(w_{1},\tau_{1}\right),\ldots ,\left(w_{T},\tau_{T}\right) \end{array}$$

where $w\in R^{V}$ and τ∈N. The vectors **w**_*t*_ are *V*-dimensional OHE representations of MBS items and the scalars *τ*_*t*_ represent the time span between two subsequent MBS items, measured in number of days. In our data set, *V* = 2774 is the vocabulary size (*i.e*. the number of unique MBS items) and *T* = 445 is the sequence length. For each sequence, **w**_*T*_ corresponds to the last MBS item before the therapy change. So the sequences can be considered *right-aligned*. Sequences shorter than *T* are zero-padded at their beginning, to prevent samples from having inconsistent representations. The first few entries of a prototypical MBS-time span sequence can look like

$$\begin{array}{c} 23\;1\;10990\;0\;23\;13\;\ldots \end{array}$$

where **w**_1_ = OHE(23), **w**_2_ = OHE(10990), **w**_3_ = OHE(23) while *τ*_1_ = 1, *τ*_2_ = 0 and *τ*_3_ = 13. The 10 most frequent MBS items of our data set are summarized in Table 1. Dealing with this kind of data, we shall keep in mind that different MBS items may have almost identical meaning. For instance, items 23 and 5020 both apply for general practitioner visits, but the second is dedicated to after-hour attendances. This can be a confounding factor that we will address in the model development process with the help of a pre-trained word embedding.

**Table 1:** Summary table of the most frequent MBS items (2.048.502 in total). Items with almost identical meaning are grouped together.

%	MBS items	Short description
0.237	10990, 10991	Management of bulk-billed services
0.187	23, 36, 5020, 5040	General practitioner attendances
0.059	73928, 73929, 73938	Collection of one or more specimens
0.037	66503, 66506, 66512, 66515, 66509	Quantitation of substances in body fluids
0.035	74995	Bulk-billing incentive
0.023	65070	Haematology
0.014	10962, 10964	Podiatric or chiropratic health service
0.014	128, 116	Consultant physician attendances
0.014	66551	Quantitation of Hba1c
0.013	105, 108	Specialist attendances

In order to cope with class imbalance, we matched positive and negatives samples by age (average on the observational time), gender, last pin state and sequence length via Coarsened Exact Matching (CEM) [24] (We used the R package cem Version 1.1.19). Matching is a non-parametric causal inference method that aims at controlling the effect of potentially confounding covariates in observational data. Via matching it is possible to prune samples such that the remaining ones have improved balance between positive and negative classes. In particular, CEM performs covariates coarsening and then creates strata of observations on which it performs exact matching, *i.e*. matched samples are retained, while unmatched ones are pruned. Table 2 is a summary table of the matched variables statistics before and after CEM matching.

**Table 2:** Summary table of the extracted data set *Pre* and *Post* matching.

		*Pre*	*Post*
# Subjects		26753	11744
Label (% Class 1)		22.02	50.00
age (years)		66.15±14.99	66.35±11.49
gender (% Female)		55.83	49.22
sequence length (# MBS items)		430.05±364.90	347.86±275.31
pin state	% ACT+NSW	39.49	35.87
	% VIC+TAS	26.15	28.73
	% WA	8.67	8.65
	% NT+SA	8.99	9.40
	% QLD	16.70	17.35

### Model description

Tangle is a two-inputs/one-output recurrent architecture which, given a set of MBS-time span sequences, returns the corresponding class probability. A pictorial representation of the model can be seen in Fig 2. In Tangle, the joint MBS-time span sequence is decoupled in two homogeneous sequences **w**_*t*_ (for *t* = 1, 3, 5, …) and *τ*_*t*_ (for *t* = 2, 4, 6, …) which are used as separate inputs of the network. The vectors **w**_*t*_ are *V*-dimensional OHE representations of MBS items. At the first layer of the network these representations are projected on a *E*-dimensional semantic space, as in Eq 4, where $x_{t}\in R^{E}$ and $W_{e}\in R^{V\times E}$.

$$\begin{array}{c} x_{t}=w_{t}W_{e} \end{array}$$ (4)

The vocabulary size *V* is defined as the number of unique MBS items observed (plus a *dummy* entry for the padding value), while the size of semantic space *E* is a free parameter of the model. In this work we tested two options for the initialization of *W*_*e*_: *uniform random* and based on the popular word-embedding GloVe [9]. More details on this second choice will be provided in the next section.

Hidden representations of the two input sequences, **x**_1_, …, **x**_*T*_ and *τ*_1_, …, *τ*_*T*_, are then achieved by two bidirectional LSTM layers [15] (see Eq 5).

$$\begin{array}{ccc} \begin{array}{c} {\vec{h}}_{x_{1}},\ldots ,{\vec{h}}_{x_{T}}=\text{LSTM}\left(x_{1},\ldots ,x_{T}\right)\\ {\overset{\leftarrow }{h}}_{x_{T}},\ldots ,{\overset{\leftarrow }{h}}_{x_{1}}=\text{LSTM}\left(x_{T},\ldots ,x_{1}\right)\\ H_{x}=\left[{\vec{h}}_{x_{1}},\ldots ,{\vec{h}}_{x_{T}},{\overset{\leftarrow }{h}}_{x_{T}},\ldots ,{\overset{\leftarrow }{h}}_{x_{1}}\right] \end{array} & & \begin{array}{c} {\vec{h}}_{\tau_{1}},\ldots ,{\vec{h}}_{\tau_{T}}=\text{LSTM}\left(\tau_{1},\ldots ,\tau_{T}\right)\\ {\overset{\leftarrow }{h}}_{\tau_{T}},\ldots ,{\overset{\leftarrow }{h}}_{\tau_{1}}=\text{LSTM}\left(\tau_{T},\ldots ,\tau_{1}\right)\\ H_{\tau }=\left[{\vec{h}}_{\tau_{1}},\ldots ,{\vec{h}}_{\tau_{T}},{\overset{\leftarrow }{h}}_{\tau_{T}},\ldots ,{\overset{\leftarrow }{h}}_{\tau_{1}}\right] \end{array} \end{array}$$ (5)

Let $H_{x}\in R^{T\times 2H}$ be the MBS bidirectional hidden representation, where *H* is the number of LSTM units. Similarly, $H_{\tau }\in R^{T\times 2H}$ is the bidirectional hidden representation of the time span sequence. For ease of notation, we define $h_{x_{t}}$ and $h_{\tau_{t}}$, for *t* = 1, …, *T* as generic 2*H*-dimensional vectors belonging to the matrices *H*_*x*_ and *H*_*τ*_, respectively.

The time span-guided neural attention mechanism adopted in Tangle can be described by the following steps.

$$\begin{array}{c} u_{x_{t}}=\text{tanh}(h_{x_{t}}W_{x}+b_{x}) \end{array}$$ (6)

$$\begin{array}{c} u_{\tau_{t}}=\text{tanh}(h_{\tau_{t}}W_{\tau }+b_{\tau }) \end{array}$$ (7)

$$\begin{array}{c} v_{t}=\lambda \;u_{x_{t}}+\left(1-\lambda \right)\;u_{\tau_{t}} \end{array}$$ (8)

$$\begin{array}{c} \alpha_{t}=\frac{\text{exp}(v_{t}W_{\alpha })}{\sum_{t=1}^{T}\text{exp}\left(v_{t}W_{\alpha }\right)} \end{array}$$ (9)

$$\begin{array}{c} \omega_{t}=h_{x_{t}}⊙\alpha_{t} \end{array}$$ (10)

Following the standard attention mechanism, $u_{x_{t}}$ and $u_{\tau_{t}}$ are hidden representations of the sequences $h_{x_{t}}$ and $h_{\tau_{t}}$ (for *t* = 1, …, *T*). These two vectors are achieved by a one-layer MLP having hyperbolic tangent activation (Eqs 6 and 7). Then, the two hidden representations are merged together in a convex combination $v_{t}\in R^{U}$ (Eq 8), where the mixin parameter λ is jointly learned at training time. This is the first novel contribution introduced by the proposed attention mechanism, with respect to the state-of-the-art.

The sequence of **v**_*t*_ is then used to obtain the weights $\alpha_{t}\in R^{2H}$ via Softmax-activated one-layer MLP (Eq 9). Finally, the attention contribution to each input element $\omega_{t}\in R^{2H}$ is expressed as the element-wise product between MBS-sequence hidden representations and the corresponding attention weights (Eq 10). Interestingly, in our case $W_{\alpha }\in R^{U\times 2H}$, the weight matrix of the Softmax layer, plays also the role of projecting the data back to a 2*H*-dimensional space, compatible with LSTM hidden representations. So, each entry of the vectors $h_{x_{t}}$ and $h_{\tau_{t}}$ (*i.e*. the output of each LSTM unit) is individually weighted. This is the second original contribution introduced by the proposed attention mechanism with respect to state-of-the-art attention. While the same scalar weight is usually associated to each of the 2*H* entries of the hidden representation **h**_*t*_, Tangle is more general as it estimates for each element in the sequence a 2*H*-dimensional attention weights vector.

The context vector $\bar{c}\in R^{E}$ is eventually computed in two steps: first by multiplying along the temporal dimension the contribution matrix

$$\begin{array}{c} \Omega^{T}={\left[\omega_{1},\ldots ,\omega_{T}\right]}^{T}\in R^{2H\times T} \end{array}$$

with the input MBS-items sequence matrix

$$\begin{array}{c} X=\left[x_{1},\ldots ,x_{T}\right]\in R^{T\times E} \end{array}$$

and secondly by average-pooling the 2*H* hidden representations (Eq 11).

$$\begin{array}{c} \bar{c}=\frac{1}{2H}\sum_{j=1}^{2H}{\left(\Omega^{T}\cdot X\right)}_{j} \end{array}$$ (11)

In the proposed architecture, the average context vector $\bar{c}$ is fed as input to a two-layers fully connected MLP and trained with Dropout [25]. The first fully connected layer has Rectified Linear Units (ReLu) activation [26], while the output probability is achieved by sigmoid *σ*(⋅) (Eq 12).

$$\begin{array}{c} \hat{y}=\sigma \left[\;\text{ReLu}\left(\bar{c}\;W_{0}+b_{0}\right)\;W_{1}+b_{1}\;\right] \end{array}$$ (12)

Tangle is trained minimizing the Cross-entropy loss (Eq 13), where *y* ∈ {0, 1} is the binary label associated with the two classes and *N* is the number of samples.

$$\begin{array}{c} L\left(y,\hat{y}\right)=-\frac{1}{N}\sum_{i=1}^{N}[y\;\text{log}\left(\hat{y}\right)+\left(1-y\right)\;\text{log}\left(1-\hat{y}\right)] \end{array}$$ (13)

Tangle is implemented in Python using Keras [27] and its source code is publicly available on GitHub at https://github.com/samuelefiorini/tangle.

### Embedding weights initialization

As previously anticipated, we need to define a protocol to initialize the embedding matrix *W*_*e*_ (see Eq 4), which is further optimized in the training phase. This matrix is used to project each MBS item in a semantic space where neighboring points correspond to MBS claims with similar meanings (see Table 1), hence working around the problem of synonym sequence elements.

We first obtained a brief textual descriptions for all the 2774 MBS items by querying the Australian Department of Health website: http://www.mbsonline.gov.au. Then, we cleaned each text corpus from punctuation and stop words. We then split the resulting descriptions in 1-grams. For instance, the word list associated to item 66551 is the following.

[quantitation, glycated, haemoglobin, performed, management, established, diabetes, item, subject, rule]

**Fig 3:**
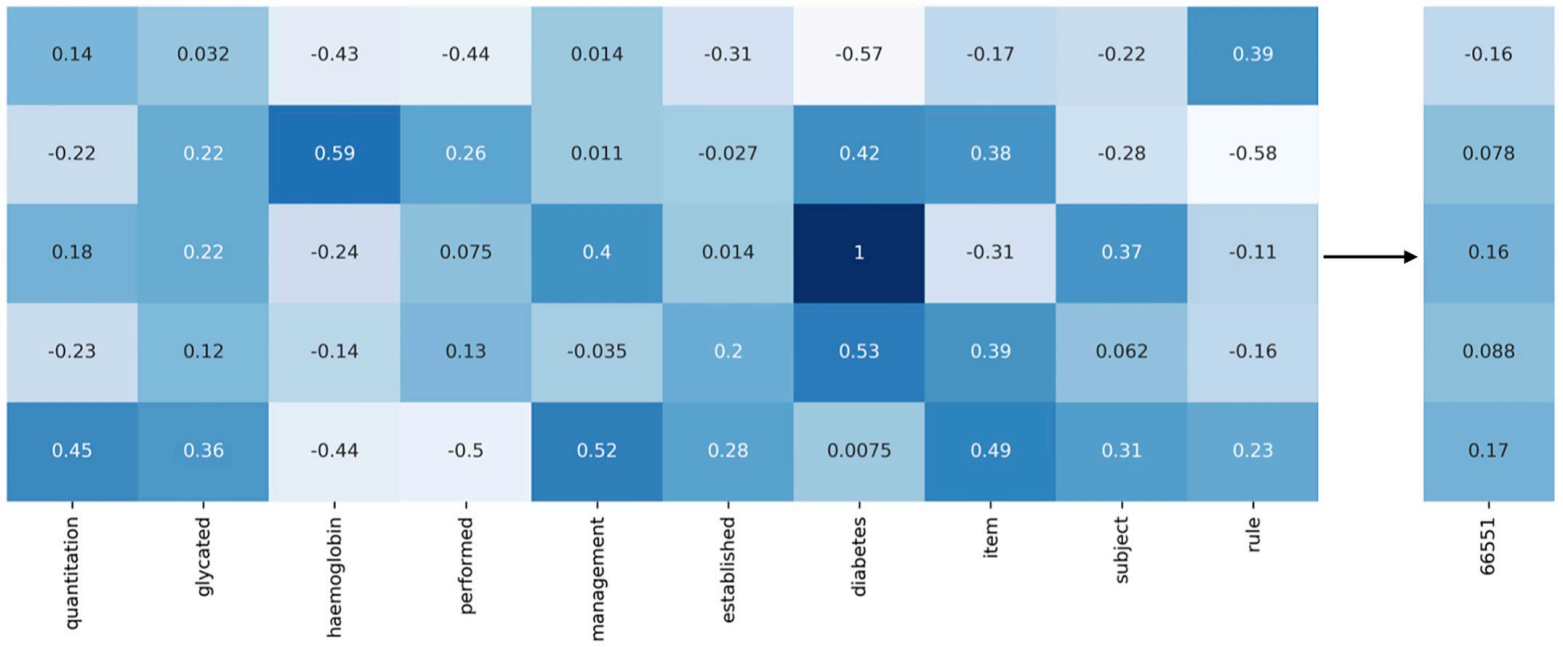
MBS item embedding. A schematic representation of our word embedding strategy to achieve meaningful representations of MBS items. Here, we consider a 10-words textual representation of the MBS item no. 66551. To each word is associated the corresponding word-embedding, which, in this picture, is a 5-dimensional vector to guarantee readability. The final representation of the considered item is achieved by averaging.

Then, we associated to each word of the list its corresponding *E*-dimensional glove.6B embedding vector, which has 4 × 10^5^ words and it is trained on *Wikipedia 2014 + Gigaword 5* data sets [9]. As of today, glove.6B comes in four increasing dimensions: 50, 100, 200, 300. In our experiments we used *E* = 50. Empirical evidences showed that larger embedding dimensions did not significantly increase Tangle prediction performance. Finally, we averaged all the single word representations, achieving an *E*-dimensional vector for each MBS item. A pictorial representation of this procedure is depicted in Fig 3. To demonstrate the effectiveness of our approach, we also tested Tangle with uniformly random initialized embedding matrix *W*_*e*_.

### Model comparison and analysis

Performance of Tangle are evaluated against three different predictive solutions.

1. *ℓ*_1_-penalized LR (see Eq 14) fitted on a *n*-BOW representation, where *n* controls the number of *n*-grams.
  $$\begin{array}{c} \hat{w}=\underset{w\in R^{d}}{\text{argmin}}\frac{1}{N}\sum_{i=1}^{N}\text{log}\left(1+e^{-y_{i}x_{i}^{T}w}\right)+\gamma \;{\left|w\right|}_{1} \end{array}$$ (14)
  In this case, **x**_*i*_ represents the *n*-BOW representation of the *i*-th patient and *d*, the dimensionality of the LR weights (**w**), depends on the number of considered *n*-grams.
2. Baseline attentionless recurrent model with bidirectional LSTM (see Fig 1).
3. State-of-the-art neural attention model with bidirectional LSTM (see Fig 2).

In order to present a fair model comparison, each tested recurrent model has the same depth, and the only difference is the attention strategy used. Performance of the tested models are evaluated via 10-split Monte Carlo cross-validation [28]. We estimated mean (*μ*) and standard deviation (*σ*) of prediction accuracy, sensitivity, specificity and Area Under the Receiver Operating Characteristics Curve (ROC AUC) [29]. The same 10 Monte Carlo sample extraction are used for every model. In each of these Monte Carlo sampling, matched data set (with *N* = 11744 samples) is split in two chunks, namely *learning* (60%) and *test* (40%). The learning set is then further split in *training* (90%) and *validation* (10%). This is led us to extract 6341 training, 705 validation and 4698 test samples for each Monte Carlo split. Training sets are used to learn the weights of every model; whereas, validation sets are used by recurrent methods to define the early stopping iteration, and by *ℓ*_1_-LR to optimize the hyperparameter *γ*, which is chosen from a grid of 10 values spanning from 10^−5^ to 1 in logarithmic scale. Model predictive performance are then evaluated on each previously unseen test samples.

## Results

We tested three increasing values of *n*: [1, 2, 3]. Empirical evidence showed that *n* = 1 yields the best performance, so results obtained with *n* ≠ 1 are not reported. The grid-search schema used to tune the regularization parameter *γ* of *ℓ*_1_-LR typically resulted in choosing $\hat{\gamma }\approx 10^{-3}$. Unpenalized LR was also tested, consistently achieving worse performance. Results of the experiments are summarized in Table 3.

**Table 3:** Summary table comparing the performance of linear and recurrent models obtained after 10 Monte Carlo cross-validation iteration. *GloVe initialization of the embedding weight matrix. **Bold** digits highlight best results.

	ROC AUC		Accuracy		Sensitivity		Specificity	
	*μ*	*σ*	*μ*	*σ*	*μ*	*σ*	*μ*	*σ*
*ℓ*_1_-LR 1-BOW	0.82	4.9e-3	0.74	4.8e-3	0.67	1.5e-2	0.81	1.1e-2
Baseline	0.81	8.4e-3	0.74	7.7e-3	0.61	4.4e-2	0.86	4.0e-2
Attention	0.84	1.1e-2	0.76	1.2e-2	0.72	4.4e-2	0.80	5.0e-2
Tangle	0.87	7.8e-3	0.78	9.9e-3	0.71	2.6e-2	0.85	2.7e-2
Baseline*	0.84	9.0e-3	0.76	9.0e-3	0.67	5.8e-2	0.84	5.2e-2
Attention*	0.86	1.2e-2	0.77	1.1e-2	0.71	3.9e-2	0.83	3.9e-2
Tangle*	**0.90**	6.0e-3	**0.82**	8.4e-3	**0.79**	3.1e-2	**0.86**	3.3e-2

**Fig 4:**
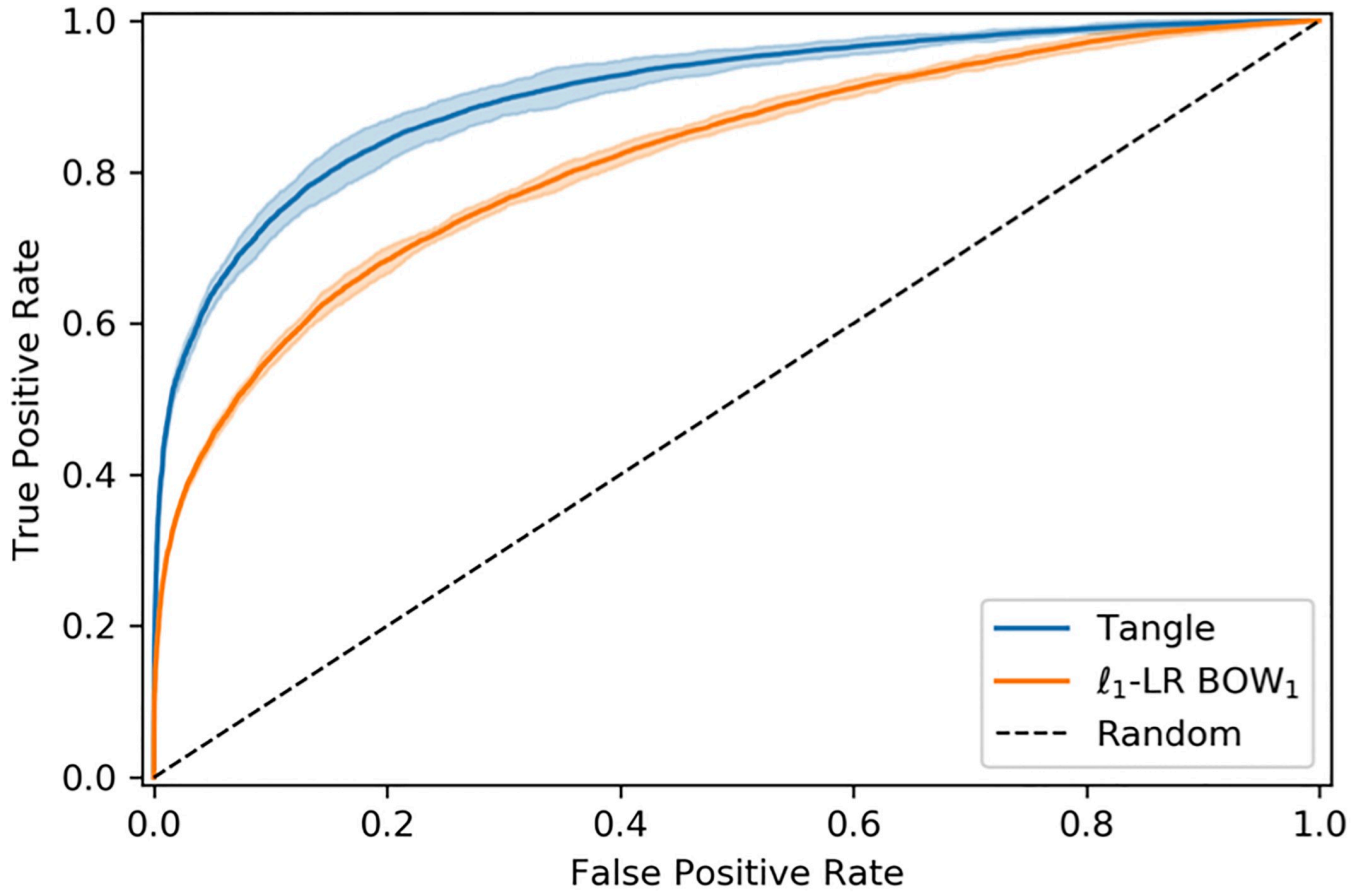
Average ROC curves. ROC curves obtained averaging the 10 Monte Carlo cross-validation iterations for best and worst method: *i.e*. Tangle and *ℓ*_1_-LR 1-BOW respectively. Shaded area corresponds to ±3*σ*, where *σ* is the standard deviation. For ease of readability, only ROC curves corresponding to best and worst performing models are shown.

**Fig 5:**
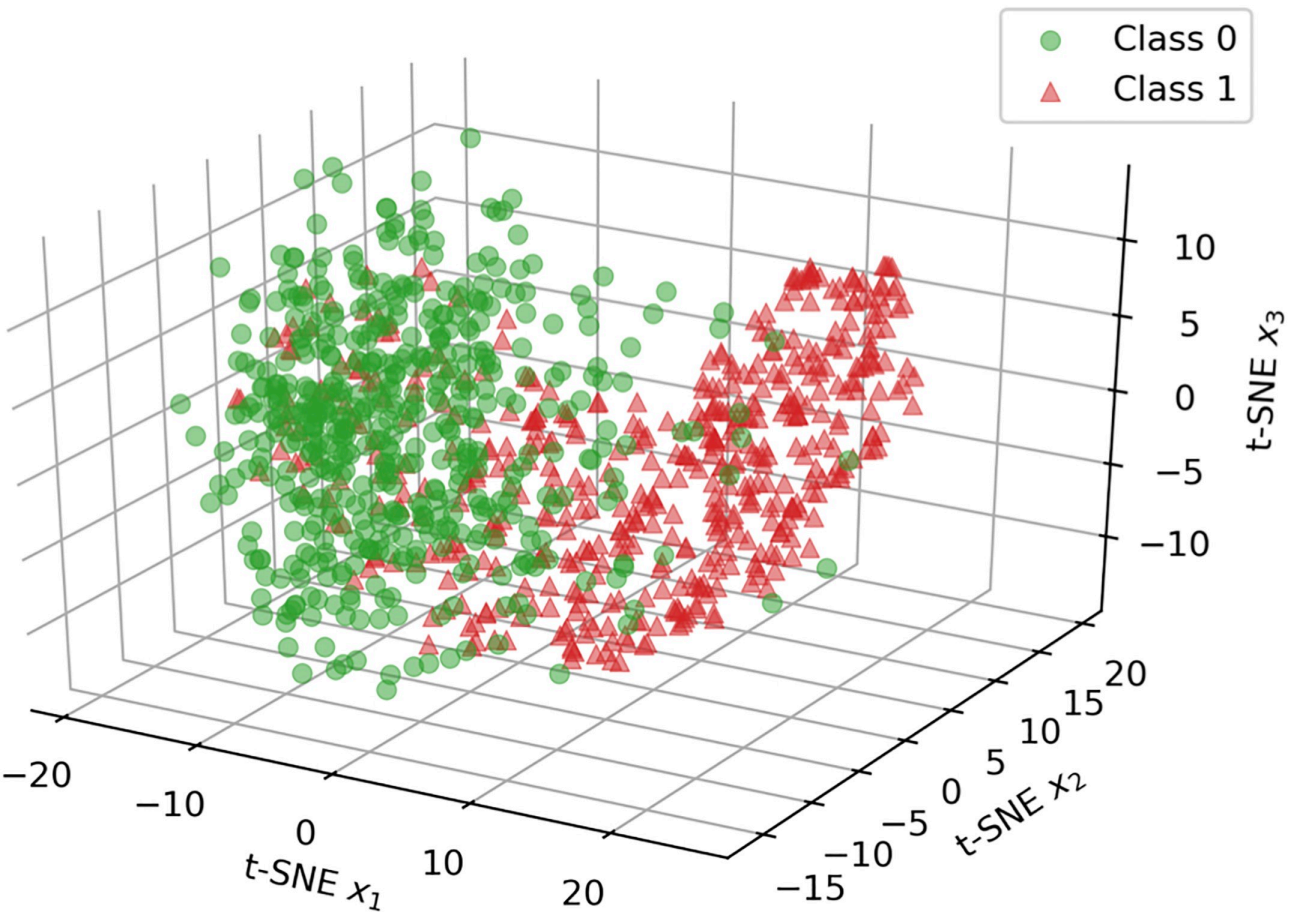
t-SNE embedding. 3D scatter-plot of a random extraction of 500 samples projected on a low-dimensional embedding, estimated by t-SNE [30], from the sample representation learned by Tangle. Samples belonging to the two classes, represented with green circles and red triangles, can be seen as slightly overlapping clusters.

Focusing on recurrent methods, Tangle outperforms baseline and state-of-the art neural attention architectures. Tangle results are also very stable across the tested Monte Carlo cross-validation procedure, in fact the corresponding standard deviation is the smallest across almost every metric. It is interesting to notice how the proposed GloVe-based initialization protocol of the embedding matrix (starred* rows in Table 3) consistently improves on every recurrent model to achieve higher ROC AUC and better classification accuracy. Therefore, we assume that the GloVe-based weight initialization ameliorates the issue of synonym MBS items. Fig 4 shows the average ROC curve obtained by Tangle and *ℓ*_1_-LR that are top and worst performing models, respectively. An intuitive visualization of the discriminative power of the representation achieved by Tangle can be seen in the 3D scatter plot of Fig 5 which was obtained by estimating a 3-dimensional t-SNE embedding [30] on the final sample representation learned by Tangle. The figure clearly shows that the learned features are able to discriminate between the two classes, explaining the good performance shown in Table 3.

**Fig 6:**
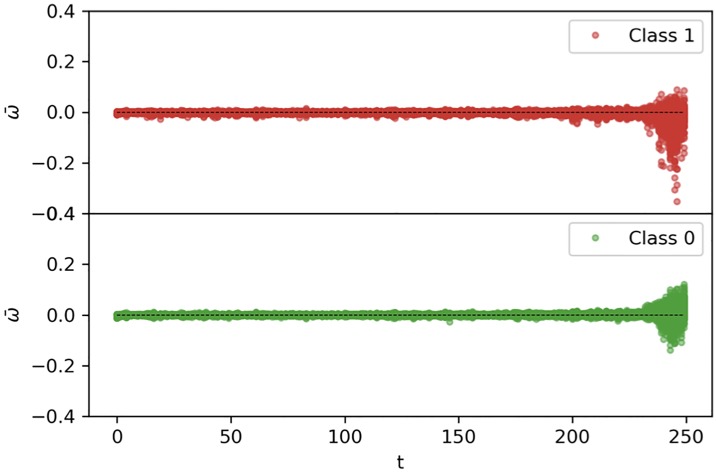
Attention contribution. Manhattan plot of the attention contribution ***ω*** estimated by Tangle on the test set. As we can see, the model correctly focuses its attention on the most recent claims, which have nonzero contributions. From this plot we can also appreciate the different representations learned for the two classes.

A visual representation of the attention contribution estimated by Tangle on the test set can be seen in the Manhattan plot of Fig 6. The horizontal axis corresponds to the MBS items sequence, while their average attention contribution ${\bar{\omega }}_{t}=\frac{1}{2H}\sum_{j=1}^{2H}\omega_{tj}$ is on the vertical axis. For ease of visualization only the last 250 MBS claims are represented. MBS-items with high attention weight are defined as the ones having $|{\bar{\omega }}_{t}|>\omega_{99}$, where *ω*_99_ corresponds to the 99-th percentile of the $|{\bar{\omega }}_{t}|$ distribution (for *t* = 1, …, *T*). From Fig 6 we can see that for both classes high attention weights are more frequently falling on the last 13 MBS-items of the sequence, which corresponds to the last 78 days (median value) before the second-line therapy transition. Moreover, we can appreciate how the specific attention weight pattern is different between the two classes.

## Discussion

The proposed model introduces two significant advances with respect to state-of-the-art recurrent models with attention. First, Tangle natively exploits time spans between two adjacent elements to guide the model attention toward the most significant events in the sequence. Secondly, the model can inherit the representational power of pre-trained word embedding in order to cope with the issue of potential synonym items in the data.

Our analysis confirms the predictive potential of recurrent models that use neural attention. We also showed that standard RNNs do not substantially outpeform simple linear models, while requiring significantly higher computational effort. On the other hand, adding the attention mechanism makes the additional computational requirement worth it, since it leads to improved performance. In addition, the proposed time span-guided attention strategy leads to even better performance, especially if coupled with pre-trained embedding initialization of the weight matrix. Overall, thanks to the available software implementation based on modern deep learning libraries, using Tangle does not require significant additional coding effort.

Another advantage of the attention mechanism is that it provides insights about which portion of the sequence might be more important. For example, in our case we found that the last 13 MBS claims, which take place in ≈ 78 days, are the most relevant for the current prediction task.

Overall, given that sensitivity and specificity of Tangle are at or above 80%, we claim that this can be the basis of an automatic alert system for patients and providers. Clearly, before Tangle can be used in practice one would have to understand at which point of the ROC curve of Fig 4 one should operate. This would require a careful analysis of the relative costs of false positives and false negative alert.

It is important to underline that there is nothing specific to DM or MBS-PBS data in Tangle. The modeling strategy and the embedding method could be applied to any problem of sequence classification, providing an easy-to-use method to represent and classify sequences composed of discrete event codes. For example, one could apply this method to the analysis of hospital data, where instead of MBS items one has ICD codes, or to more complex data sets, such as the Electronic Health Record collection MIMIC-III [31], that contains clinical codes as well as clinical measures and doctors’ notes.
